# Rewiring E2F1 with classical NHEJ via APLF suppression promotes bladder cancer invasiveness

**DOI:** 10.1186/s13046-019-1286-9

**Published:** 2019-07-08

**Authors:** Christin Richter, Stephan Marquardt, Fanghua Li, Alf Spitschak, Nico Murr, Berdien A. H. Edelhäuser, George Iliakis, Brigitte M. Pützer, Stella Logotheti

**Affiliations:** 1 Institute of Experimental Gene Therapy and Cancer Research, Rostock University Medical Center, Schillingallee 69, 18057 Rostock, Germany; 2 Institute of Medical Radiation Biology, University of Duisburg-Essen Medical School, Essen, Germany; 3 Department Life, Light and Matter of the Interdisciplinary Faculty at Rostock University, Rostock, Germany

**Keywords:** E2F1, miR-888, APLF, Bladder cancer, Non-homologous end-joining

## Abstract

**Background:**

Bladder cancer progression has been associated with dysfunctional repair of double-strand breaks (DSB), a deleterious type of DNA lesions that fuel genomic instability. Accurate DSB repair relies on two distinct pathways, homologous recombination (HR) and classical non-homologous end-joining (c-NHEJ). The transcription factor E2F1 supports HR-mediated DSB repair and protects genomic stability. However, invasive bladder cancers (BC) display, in contrast to non-invasive stages, genomic instability despite their high E2F1 levels. Hence, E2F1 is either inefficient in controlling DSB repair in this setting, or rewires the repair apparatus towards alternative, error-prone DSB processing pathways.

**Methods:**

RT-PCR and immunoblotting, in combination with bioinformatics tools were applied to monitor c-NHEJ factors status in high-E2F1-expressing, invasive BC versus low-E2F1-expressing, non-invasive BC. In vivo binding of E2F1 on target gene promoters was demonstrated by ChIP assays and E2F1 CRISPR-Cas9 knockdown. MIR888-dependent inhibition of APLF by E2F1 was demonstrated using overexpression and knockdown experiments, in combination with luciferase assays. Methylation status of *MIR888* promoter was monitored by methylation-specific PCR. The changes in invasion potential and the DSB repair efficiency were estimated by Boyden chamber assays and pulse field electrophoresis, correspondingly.

**Results:**

Herein, we show that E2F1 directly transactivates the c-NHEJ core factors Artemis, DNA-PKcs, ligase IV, NHEJ1, Ku70/Ku80 and XRCC4, but indirectly inhibits APLF, a chromatin modifier regulating c-NHEJ. Inhibition is achieved by miR-888-5p, a testis-specific, X-linked miRNA which, in normal tissues, is often silenced via promoter methylation. Upon hypomethylation in invasive BC cells, *MIR888* is transactivated by E2F1 and represses APLF. Consequently, E2F1/miR-888/APLF rewiring is established, generating conditions of APLF scarcity that compromise proper c-NHEJ function. Perturbation of the E2F1/miR-888/APLF axis restores c-NHEJ and ameliorates cell invasiveness. Depletion of miR-888 can establish a ‘high E2F1/APLF/DCLRE1C’ signature, which was found to be particularly favorable for BC patient survival.

**Conclusion:**

Suppression of the ‘out-of-context’ activity of miR-888 improves DSB repair and impedes invasiveness by restoring APLF.

**Electronic supplementary material:**

The online version of this article (10.1186/s13046-019-1286-9) contains supplementary material, which is available to authorized users.

## Background

Urothelial carcinomas constitute the most common bladder cancer type. Their majority is superficial at diagnosis (non-muscle-invasive bladder carcinoma, NMIBC), presenting good prognosis upon surgical resection and localized therapies. However, those invading the muscle wall (muscle-invasive bladder carcinoma, MIBC) are metastasis-prone and treatment-refractory [1]. The strikingly different outcomes rely on the fact that MIBCs exhibit distinct molecular characteristics, which if unveiled, could be appropriately manipulated towards precision management of advanced disease stages. One major difference is that MIBCs, in comparison to NMIBCs, show an error-prone repair of double-strand breaks (DSB) [2], one of the most dangerous types of DNA lesions.

DSB repair occurs via two major mechanisms: homologous recombination (HR), which uses a homologous DNA template from sister chromatids, or classical non-homologous end-joining (c-NHEJ), which is faster and religates any type of ends without requiring homologous sequences [3]. Due to its high repair rate, c-NHEJ largely suppresses chromosome translocations, while sequence alterations at the junction remain possible. It has now become clear that when either c-NHEJ or HR is inactivated, an alternative form of end-joining (alt-EJ) takes over. Alt-EJ operates with speed and fidelity markedly lower than c-NHEJ, thus increasing chances for chromosome translocations and more extensive sequence alterations at the junction. Consequently, alt-EJ serves as a backup for eliminating highly cytotoxic DSBs, but the repair is performed in an error-prone and mutagenic manner [4, 5]. A shift of DSB repair towards mutagenic NHEJ increases genomic instability and tumor heterogeneity, thereby favoring selection of therapy-resistant cancer phenotypes [6].

In MIBCs, c-NHEJ inactivation is followed by a preference for alt-EJ that exacerbates genomic instability [2]. Invasive BC tumors display alt-EJ which leads to large deletions, while in non-invasive BC tumors the still functional c-NHEJ machinery repairs DSBs more accurately [7]. Given that accuracy relies on the coordinated and sophisticated interaction of core c-NHEJ factors, it is plausible that this switch is a consequence of deregulated expression and/or activity of one or more of the factors within this machinery. Nonetheless, thus far, drivers of this reported c-NHEJ dysregulation in BC remain largely unknown.

To function accurately, the c-NHEJ machinery requires the Ku heterodimer, which comprises a 70 kDa (Ku70, encoded by *XRCC6*) and an 80 kDa (Ku80, encoded by *XRCC5*) subunit that bind to DSBs. It also requires the DNA-dependent protein kinase catalytic subunits (DNA-PKcs, encoded by *PRKDC*), NHEJ1 (also known as XLF, PAXX, or Cernunnos, encoded by *NHEJ1*), XRCC4, and DNA ligase IV (encoded by *LIG4*) which are recruited by Ku to the lesion site. DNA-PKcs promote DNA end processing by the nuclease Artemis (encoded by *DCLRE1C*), whereas XRCC4-LIG4 and NHEJ1 facilitate the final step of DNA ligation. Loss-of-function mutations of the above-mentioned factors in rodent models and cancer patients lead to defective DSB repair [8].

A crucial step is the recruitment of APLF (Aprataxin and Polynucleotide kinase Like Factor) by Ku80. APLF is a chromatin modifying protein of increasing importance for c-NHEJ [9]. It encompasses distinct functional domains, through which it acts as an endo/exonuclease and a generic histone chaperone. APLF interacts with the XRCC4/LIG4 complex through the N-terminal forkhead-associated domain [10], while it binds to Ku80 via its middomain [11], collectively generating a scaffold to form c-NHEJ complex and facilitate DNA ligation [12]. Through its C-terminal domain, APLF exhibits histone chaperone activity, mediating recruitment to the DNA damage site, chromatin assembly, binding to core histones, and nucleosome disassembly [13]. Disruption of the interactions between APLF and either Ku80 or XRCC4-LIG4 compromises the assembly and activity of Ku complexes and reduces DSB repair rates [9].

E2F1, the prototype member of the E2F family of transcription factors, enhances DSB repair [14], constituting a bona fide factor of genomic stability. In particular, it controls HR-mediated DSB repair by transactivating HR-related genes [15], recruiting repair factors [16], and participating in repair complexes with HR-mediators at DSB sites [17]. However, while E2F1 constitutes the “final frontier” of the G1-to-S phase boundary that preserves cellular homeostasis, this factor switches duties during cancer progression and is engaged to pathways favoring invasiveness, therapy resistance, and metastasis [18]. High E2F1 levels correlate with progression from superficial to MIBC stages [19]. Additionally, E2F1 is a hub in core regulatory networks of BC invasiveness and can also be part of molecular signatures which are prognostic for patient survival [20]. The existence of genomic instability in MIBCs, despite E2F1 abundance, implies that E2F1 is either inefficient of controlling DSB repair or rewires DSB repair networks towards error-prone repair which eventually facilitates invasiveness. However, it remains unclear how E2F1 turns from friend to foe of genomic stability. In light of these observations, we considered that an E2F1-mediated impairment of c-NHEJ could provide a mechanistic basis for the reported error-prone repairing in MIBCs [2]. To test this hypothesis, we investigated the impact of E2F1 on c-NHEJ factors in conjunction with the invasive characteristics of MIBCs. To our knowledge, the systemic effect of E2F1 on c-NHEJ in the context of MIBC has not been addressed before.

## Methods

### Cell culture and treatments

Human bladder cancer cell lines were maintained and treated as described in Additional file 1.

### RNA and DNA isolation, TaqMan MicroRNA assay, RT-PCR, qPCR and methylation-specific PCR

RNA isolation, reverse transcription, RT-PCR and qPCR were performed as described earlier [21, 22]. The primer sequences are shown in Additional file 1, Table S1. In general, large and small RNA was extracted using the NucleoSpin® miRNA kit (MACHEREY-NAGEL). MicroRNA expression levels were quantitated using TaqMan® MicroRNA single assays and 7900HT Fast Real-Time PCR System (Applied Biosystems). Expression analysis was performed using the comparative CT method with RNU6B as endogenous control. For semi-quantitative PCR, 1 μg of RNA was reverse transcribed using First Strand cDNA Synthesis Kit (Thermo Scientific). The cDNA was added to peqGOLD Hot Start Mix Y (2×) and amplified with specific primers in a T100™ Thermal Cycler (Bio-Rad). For qRT-PCR, cDNA was added to iTaq™ Universal SYBR Green Supermix and analyzed using CFX96 Touch™ Real-Time PCR Detection System (Bio-Rad). Relative gene expression was calculated by comparative CT method using actin and GAPDH for normalization. Primers for hsa-miR-888 (RT/TM002212) and RNU6B (RT/M001093) were purchased from Applied Biosystems. Genomic DNA isolation, bisulfite treatment, and methylation-specific PCR are described in Additional File 1.

### Immunoblotting

Immunoblots were performed as previously described [23] using antibodies against E2F1 (#3742), Artemis (D708V, #13381), DNA Ligase IV (D5N5N, #14649), DNA-PKcs (# 4602), Ku70 (D10A7, #4588), Ku80 (C48E7, #2180), XLF (#2854) from Cell Signaling; actin (ac-15, Sigma); APLF (ab196502, abcam) and XRCC4 (C-4, sc-271087, Santa Cruz).

### Plasmid constructs, adeno- and lentiviral vectors and CRISPR-Cas9 knockdown cells

Plasmid constructs, viral vectors and CRISPR-Cas9 knockdown are described in Additional File 1.

### Chromatin immunoprecipitation and luciferase reporter assays

Experiments were performed as described in Additional file 1.

### Wound healing and invasion assays

Wound healing and Boyden chamber assays on cell lines and their derivative clones were performed as previously described [23].

### Pulse field gel electrophoresis assays (PFGE)

Cells were exposed to 20 Gy X-rays and incubated for repair. After different incubation periods, cells were trypsinized, resuspended in serum-free medium (20 mM Hepes, 5 mM NaHCO_3_) at a concentration of 6 × 10^6^ cells/ml and mixed with equal volume of 1% low-melting agarose (Bio-Rad, Munich, Germany). Agarose plugs (5 mm long), were prepared and lysed as described [24].

PFGE was performed in gels cast with 0.5% agarose (Bio-Rad), in 0.5X TBE at 8 °C for 40 h. The electric field was pulsed at 50 V (1.25 V/cm) for 900 s in the forward direction and 200 V (5.00 V/cm) for 75 s in reverse direction. Then, gels were stained with 1.6 μg/ml ethidium bromide and imaged using a fluoroimager (Molecular Dynamics Typhoon 9400, GE Healthcare, Freiburg, Germany). The fraction of DNA released (FDR) was analyzed by ImageQuant 5.2 (GE Healthcare, Freiburg, Germany) and used to calculate the equivalent of Gy dose (Deq) [25].

### Bioinformatics and statistical analyses

Bioinformatics analyses are described in Additional file 1. Unless stated otherwise, all experiments were carried out in triplicates and values were expressed as mean ± standard deviation (SD). Statistical analyses of in vitro experiments were performed using paired Student’s t-test. *P*-values less than 0.05 were considered significant. All statistical tests were two-sided. Statistical significance of the survival curves was estimated by log-rank test. Bonferroni correction was used for multiple comparison adjustment (Additional file 1, Table S3).

## Results

### Expression of c-NHEJ factors is deregulated in invasive, high-E2F1 bladder cancer

**Fig. 1:**
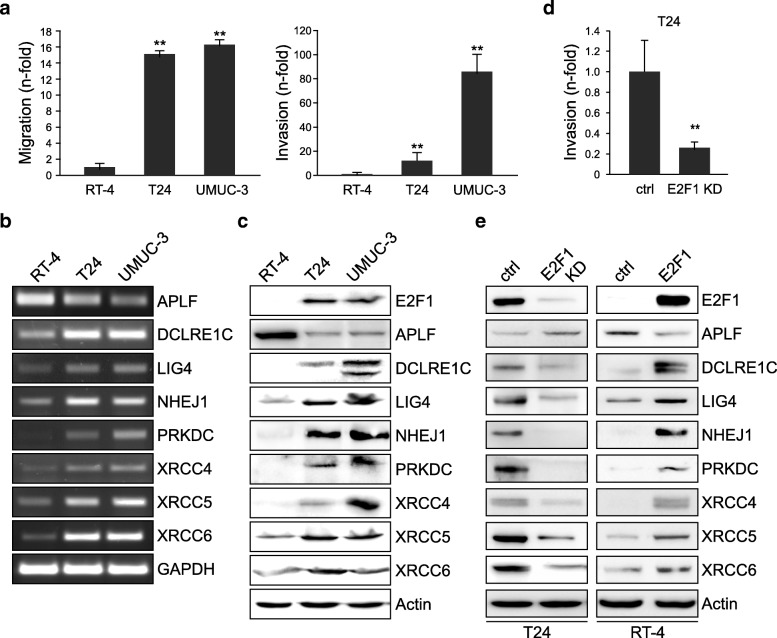
Levels of c-NHEJ factors are deregulated in high E2F1-expressing invasive BC cells. **a** Cell migration (*left*) and invasion assays (*right*) in high E2F1-expressing T24 and UMUC-3 cells versus the low E2F1-expressing RT-4 cells. RT-4 values are set as 1. **b** Semi-quantitative PCR of c-NHEJ factors in RT-4, T24 and UMUC-3 cells. **c** Immunoblot depicting endogenous levels of c-NHEJ factors in RT-4, T24, and UMUC-3 cells. **d** Matrigel assays in E2F1-depleted T24 cells compared to the control. **e** Western blot analyses of c-NHEJ factors in T24 and RT-4 cells after knockdown or overexpression of E2F1, respectively, versus their corresponding controls. Asterisks denote statistically significant (** *p* < 0.01) changes

Superficial-to-invasive BC progression is linked to E2F1 upregulation [19], and invasive BC cell lines express, in contrast to non-invasive cells, high E2F1 levels [20]. Consistently, the BC cell lines T24 and UMUC-3, which exhibit a higher migratory (Fig. 1a, left) and invasive (Fig. 1a, right) capacity show significantly elevated E2F1 protein levels compared to RT-4 [20] derived from superficial low-grade bladder tumors [26, 27]. We have recently shown that addition of E2F1 in RT-4 enhances the cells’ invasive potential, while downregulation of E2F1 in UMUC-3 suppresses this capacity [20]. Based on these findings, we investigated a possible association between E2F1 and the expression of the NHEJ machinery factors in conjunction with the observed aggressive cell behavior. Both endogenous mRNA (Fig. 1b) and protein (Fig. 1c) levels of DCLRE1C (Artemis), LIG4 (ligase IV), PRKDC (DNA-PKcs), NHEJ1, XRCC4, XRCC5 (Ku80), and XRCC6 (Ku70) are co-elevated with E2F1 in invasive cell lines. In contrast, APLF is significantly downregulated in T24 and UMUC-3 versus less-invasive RT-4 (Fig. 1b, c).

To estimate if c-NHEJ factors’ expression correlates with E2F1-driven invasiveness, we transiently knocked-down E2F1 in T24 cells using shRNA and subsequently performed Boyden chamber assays and immunoblotting. Indeed, we observed that the significantly decreased invasiveness of E2F1-depleted cells versus their controls (Fig. 1d) is accompanied by downregulation of DCLRE1C, LIG4, NHEJ1, PRKDC, XRCC4, and XRCC5/XRCC6, and upregulation of APLF. Vice versa, addition of E2F1 in non-invasive RT-4 cells leads to increased expression of these proteins while APLF is reduced (Fig. 1e). Collectively, while the expression of all core c-NHEJ factors tested increases in response to high E2F1, APLF expression is inversely related to E2F1.

### E2F1 directly transactivates c-NHEJ core factors, but downregulates APLF indirectly

**Fig. 2:**
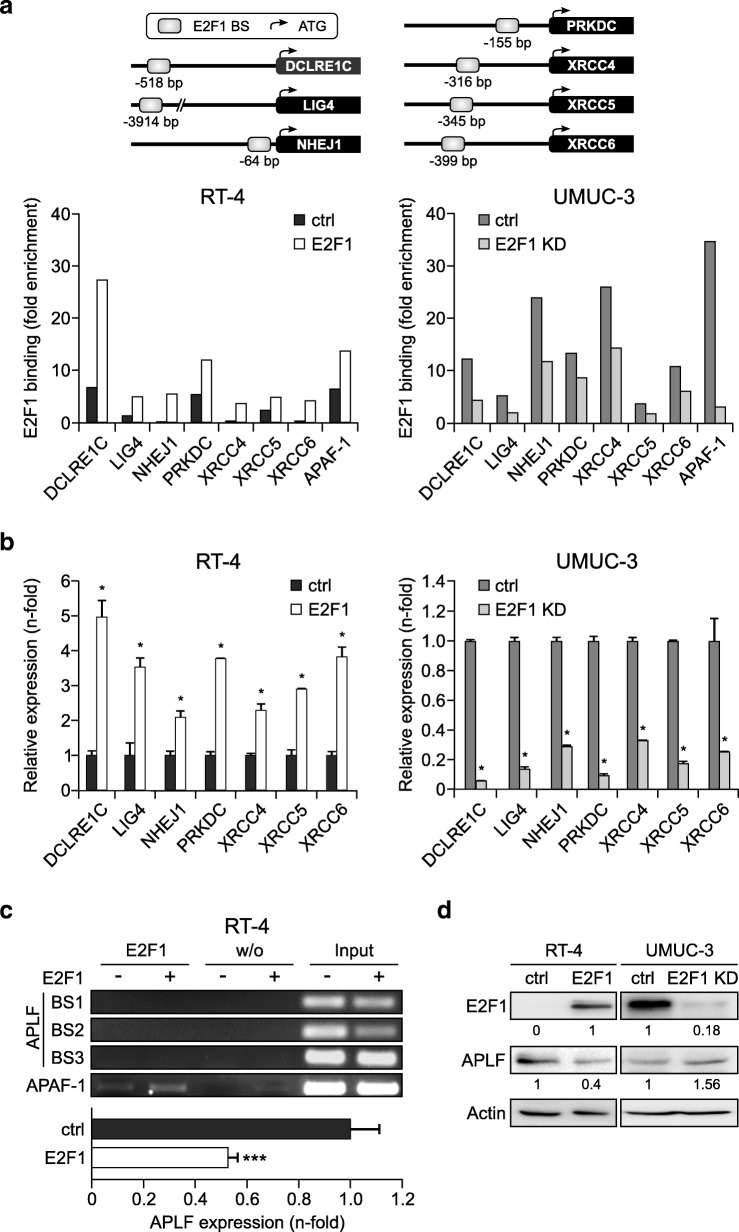
E2F1 transactivates c-NHEJ factors directly, but downregulates APLF indirectly. **a** *Top:* Schematic representation of the positions of the E2F1 responsive elements on the promoter of depicted c-NHEJ genes relative to the ATG codon. Primers were designed that encompass these sites, and the ability of E2F1 to directly interact with the corresponding elements in vivo was demonstrated by ChIP using specific E2F1 monoclonal antibody, followed by genomic PCR analysis. *Bottom:* ChIP assays, using anti-E2F1 antibody in RT-4 after ectopic E2F1 expression (*left*) and in stable UMUC-3 E2F1 knockdown cells (E2F1 KD) versus their controls (ctrl, *right*) demonstrated functional binding sites in the promoters of *DCLRE1C*, *LIG4*, *NHEJ1*, *PRKDC*, *XRCC4*, *XRCC5,* and *XRCC6* genes. Mock IPs of the cell lysates were used as a background control and binding was expressed as fold-enrichment of each sample relative to mock IP controls. The E2F1 binding site of the *APAF-1* promoter was used as a positive control for functional E2F1 binding sites. **b** qPCR mRNA levels of above-mentioned c-NHEJ factors in BC cell lines with (*left*) and without E2F1 (*right*). Asterisks indicate statistically significant (* *p* < 0.05) changes in comparison to the controls. **c** *Top:* ChIP assays in RT-4-E2F1 cells versus RT-4-ctrl for the *APLF* promoter regions − 673 to − 472 (BS1), − 492 to − 316 (BS2), and − 67 to + 75 (BS3) bps relative to the ATG codon showed no E2F1 binding in any of the three regions. Mock IPs incubated without antibody (w/o Ab) were used as a negative control, while input was used as positive control. The *APAF-1* promoter was used as an E2F1 binding control. *Bottom*: qPCR in RT-4-E2F1 cells versus RT-4-ctrl cells for APLF (*** *p* < 0.001). **d** Detection of E2F1 and APLF protein levels in indicated BC cells. Band quantification was performed by imageJ software

We next examined whether the c-NHEJ factors are subject to E2F1 transcriptional regulation. In silico analysis revealed putative E2F1-responsive binding motifs in the *NHEJ1*, *DCLRE1C*, *LIG4*, *PRKDC*, *XRCC4*, *XRCC5*, and *XRCC6* promoters at the positions indicated in Fig. 2a. E2F1 binding to these sites was tested via ChIP assays in the least invasive RT-4 cells upon exogenous E2F1 expression and in the most invasive UMUC-3 cells upon stable E2F1 knockdown. The results showed enrichment of E2F1 binding to the promoter regions of all these c-NHEJ core factors upon E2F1 addition (Fig. 2a, left), while binding was drastically reduced in E2F1-ablated cells compared to the controls (Fig. 2a, right). In agreement, transcription of the corresponding mRNA is augmented upon E2F1 addition in RT-4 cells (Fig. 2b, left), whereas it is diminished upon stable E2F1 depletion in UMUC-3 (Fig. 2b, right).

Moreover, several putative E2F1 binding sites were detected in the APLF promoter between positions − 503 to + 50 bps relative to the ATG codon. However, ChIP assays revealed that E2F1 does not bind to these motifs under in vivo conditions (Fig. 2c, top). Instead, APLF mRNA decreased through E2F1 upregulation (Fig. 2c, bottom). Furthermore, immunoblots with anti-APLF showed a clear APLF downregulation in response to E2F1 addition in RT-4 cells, whereas APLF expression markedly increased in E2F1-ablated UMUC-3 (Fig. 2d). These data collectively demonstrate that E2F1 transactivates core factors of the c-NHEJ machinery, while it inhibits *APLF* gene expression via non-transcriptional regulation.

### APLF or DCLRE1C levels in E2F1-expressing bladder tumors are associated with patient outcomes

**Fig. 3:**
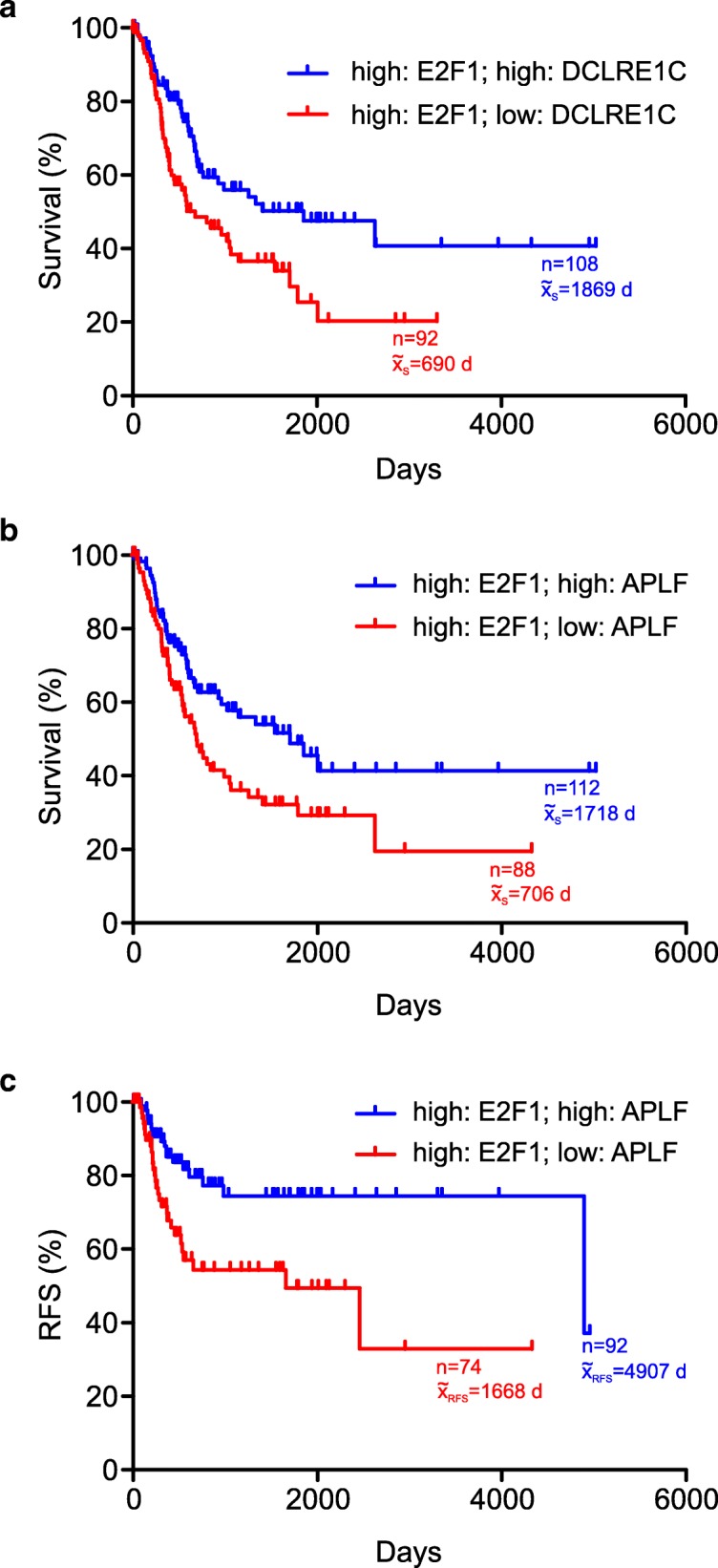
DCLRE1C and APLF levels in E2F1-expressing tumors are correlated with patient survival. **a, b** Overall survival (OS) curves of BC patients with (**a**) high E2F1/high DCLRE1C (blue) versus high E2F1/low DCLRE1C (red) mRNA levels, (**b**) high E2F1/high APLF (blue) versus high E2F1/low APLF mRNA levels (red). **c** Recurrence-free survival (RFS) of BC patients with high E2F1/high APLF (blue) versus high E2F1/low APLF mRNA levels (red)

As a crucial regulator of key events in the metastatic cascade, increased abundancy of E2F1 is associated with aggressiveness and unfavorable prognosis in many cancers such as BC [18, 28]. In this regard, we have recently shown that E2F1-responsive factors which are causative for cancer progression associate with poor patient survival when combined with high coexpression of E2F1 [20, 29]. Since increased levels of this transcription factor cause deregulation of core c-NHEJ factors, we examined which of these changes are relevant for progressive disease and survival outcome in a high E2F1 context (reflecting primary BC tumors with the potential to grow invasively) and performed Kaplan-Meier analyses of RNA-Seq data from TCGA cohorts of BC patients with high E2F1-expressing tumors, for each individual c-NHEJ factor. Our findings show that changes in the mRNA levels of the core c-NHEJ factors LIG4, NHEJ1, PRKDC, XRCC4, XRCC5, and XRCC6 do not produce significantly different outcomes relative to the rest of the BC patient population (Additional File 1, Table S3). This is in line with an earlier study, reporting a lack of correlation between invasiveness/error-prone joining and LIG4, PRKDC, XRCC4, XRCC5, or XRCC6 protein levels in invasive BC tumors [7]. In contrast, our analysis indicated that APLF and DCLRE1C are the clinically relevant determinants which promote patient survival. In particular, DCLRE1C mRNA levels were able to discriminate between better and worse (Fig. 3a) overall survival (OS) in high E2F1 tumors. In light of this, high DCLRE1C in high E2F1 MIBC cell lines is consistent with a favorable survival signature. Furthermore, we observed that patients with high E2F1 BC tumors exhibit both, better overall (Fig. 3b) and recurrence-free survival (Fig. 3c) upon increased APLF mRNA expression, whereas both worsen when APLF levels are low. This inverse correlation between E2F1 and APLF in patients with poor survival and disease relapse is reminiscent to the expression pattern of invasive BC cell lines shown in Fig. 1b. Thus, E2F1-induced reduction of APLF in invasive BC cells emerges as the crucial event during malignant progression.

### E2F1 inhibits APLF via transactivation of *MIR888*

Given the clinical importance of the high E2F1/low APLF correlation in BC, we sought to mechanistically dissect how APLF is suppressed in E2F1-expressing BC cells. A common class of negative gene regulators includes, but is not limited to, miRNAs. MiRNA genes synthesize 16 to 27 nucleotide-long non-coding RNA molecules which bind to 3’UTR of mRNAs and either induce their degradation or inhibit translation [30]. We therefore examined whether APLF is post-transcriptionally inhibited by miRNAs in high E2F1-expressing cells.

**Fig. 4:**
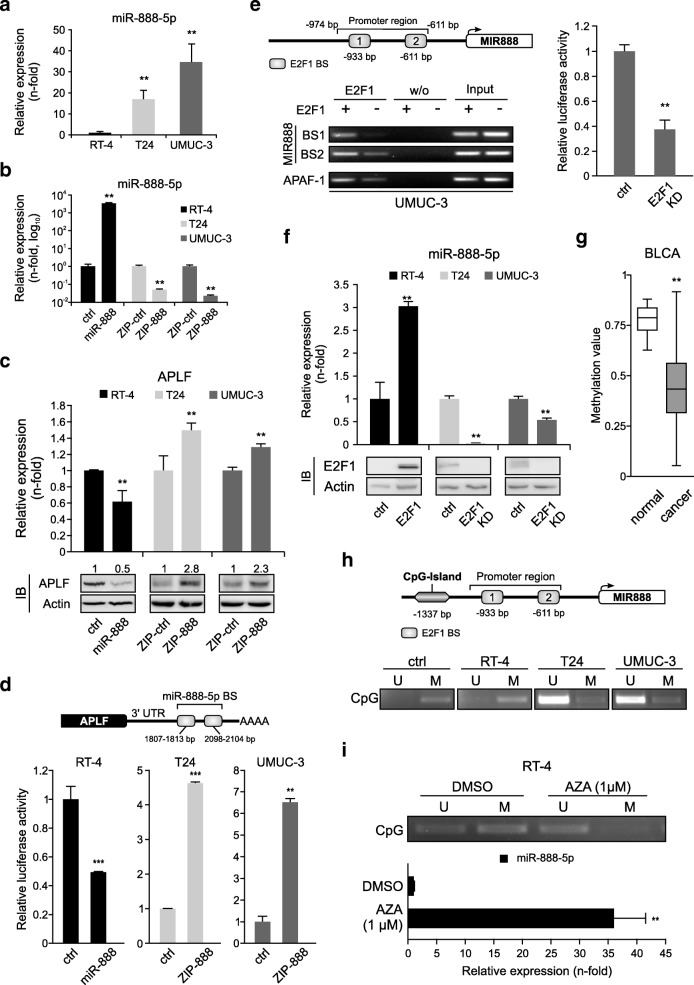
E2F1 accesses the hypomethylated *MIR888* and transactivates the APLF inhibitor miR-888-5p in invasive BC. **a** Validation of endogenous miR-888-5p transcript levels in RT-4, T24 and UMUC-3 cells. MiRNA expression in RT-4 cells was set as 1. **b, c** qPCR of miR-888-5p (b) and APLF levels (c, *top***)** in RT-4 expressing mir-888-5p (black), in T24.ZIP-888 (light grey) and in UMUC-3.ZIP-888 (dark grey) versus controls. Immunoblots (IB) of APLF are shown *below* with actin as loading control. **d** Scheme of the APLF 3’UTR with predicted miR-888-5p binding sites (BS). Reporter assays with above-mentioned cell lines co-transfected with the 3’UTR luciferase construct. Controls were set as 1. **e** *Top*: Scheme of the *MIR888* promoter region with two predicted E2F1 binding sites. *Bottom*: ChIP assays for *MIR888* promoter in E2F1-depleted UMUC-3 cells using anti-E2F1 antibody. Mock IPs (w/o) of the samples were used as a negative control, while input was used as positive control. The *APAF-1* promoter was used as an E2F1 binding control. *Right*: The promoter activity was measured using a luciferase assay and the control was set as 1. **f** Quantification of miR-888-5p in indicated BC cell lines after overexpression or knockdown (KD) of E2F1. E2F1 protein levels were validated using immunoblots (*bottom*), using actin as loading control. **g** Methylation status of *MIR888* promoter region ranging 2000 bps upstream of TSS in Bladder Urothelial Carcinoma (BLCA). **h** *Top*: *MIR888* promoter region with the CpG island in proximity to the E2F1 binding sites. *Bottom*: Methylation-specific PCR in RT-4, T24, UMUC-3 cells using primers that discriminate between the unmethylated (U) and methylated (M) CpG island upstream of *MIR888* TSS. The human methylated DNA standard (ctrl) was used as positive control. **i** Methylation-specific PCR for *MIR888* CpG island in AZA-treated RT-4 cells (*top*) and validation of miR-888-5p levels (*bottom*). Asterisks indicate statistically significant (** *p* < 0.01, *** *p* < 0.01) changes

The 3’UTR of APLF is predicted to be targeted with a high miTG score [31] by miR-888-5p, an oncomiR which is upregulated across several cancer types [32–37]. As shown in Fig. 4a, miR-888-5p levels are elevated in invasive versus less-invasive BC cells and this is in inverse correlation with APLF expression (Fig. 1b, c), providing hints that miR-888-5p upregulation inhibits APLF. To dissect the miR-888-5p/APLF interaction, we established a study system, where miR-888-5p is overexpressed in RT-4 (Fig. 4b, black bars) via transient transfection with miR-888 expression plasmid, while it is inhibited in T24 and UMUC-3 cells through stable expression of miR-888-5p antagomiR (T24.ZIP-888 and UMUC-3.ZIP-888) (Fig. 4b, light and dark grey bars). While APLF mRNA and protein levels decreased in response to miR-888-5p upregulation, specific repression of this miRNA leads to a substantial APLF increase (Fig. 4c).

To further validate APLF targeting by miR-888-5p, a luciferase construct carrying the 3’UTR of human APLF downstream of the reporter gene (3’UTRAPLF-Luc) was transiently co-transfected with miR-888 expression plasmid in RT-4 cells. This resulted in a significant decrease of luciferase activity compared to the control (Fig. 4d, black bars). Contrariwise, the luciferase signal considerably increased in T24.ZIP-888 and UMUC-3.ZIP-888 cells transfected with the 3’UTRAPLF-Luc plasmid versus the controls (Fig. 4d, light grey and dark grey bars). Of note, APLF is the only c-NHEJ factor sensitive to miR-888-5p. In particular, miR-888-5p is not predicted by the DIANA-microT-CDS platform [31] to target either E2F1 or the E2F1-regulated c-NHEJ factors and, consequently, its knockdown has a negligible impact on their mRNA levels (Additional File 2, Figure S1), demonstrating high specificity for APLF.

Since the miR-888-5p levels in T24 and UMUC-3 versus RT-4 (Fig. 4a) correlate with E2F1 expression in these cell lines (Fig. 1c), and two putative E2F1 binding motifs were predicted at − 933 and − 611 bps upstream of the miRNA TSS (Fig. 4e, top), we hypothesized that *MIR888* itself constitutes an E2F1 target. ChIP assays in stable UMUC-3.E2F1-knockdown cells confirmed its specific binding to the responsive elements (Fig. 4e, bottom). Moreover, luciferase assays in E2F1-depleted cells using the *MIR888* upstream region that encompasses both binding motifs revealed a significantly reduced promoter activity compared to UMUC-3 control (Fig. 4e, right). Consistently, E2F1 addition in less invasive BC cells substantially enhanced, and downregulation of E2F1 in invasive cells diminished miR-888-5p expression (Fig. 4f). Thus, the APLF blockade in high E2F1-expressing MIBC is achieved via E2F1-mediated transcriptional upregulation of the APLF inhibitor miR-888-5p.

### E2F1-induced transactivation of the testis-specific *MIR888* is enabled by promoter hypomethylation

E2F1 and APLF are normally expressed in several anatomical sites (data mined from Human Protein Atlas, www.proteinatlas.org). In contrast, studies of *MIR888* in a wide-range of normal human tissues, including bladder, have revealed a testis-specific expression pattern [34, 38]. In agreement, data mining from MIRIAD database [39] revealed that miR-888-5p expression is normally very low and strictly restricted to a few tissues in addition to testis, like cerebellum, heart, and kidney (Additional File 3, Figure S2), suggesting that in the majority of normal tissues, *MIR888* is silenced. Expression of tissue-restricted genes during malignant transformation, a process known as ‘ectopic activation’, is achieved via epigenetic reprogramming [40]. With this in mind, we turned our attention to possible epigenetic events which could keep *MIR888* under strict regulation and might have been reversed in BC.

Given that gene methylation is dysregulated in BC [41], we monitored the methylation status of the *MIR888* promoter. Data mined from the Human Disease Methylation Database [42] showed that, in urothelial BC, *MIR888* is hypomethylated compared to corresponding normal tissue (Fig. 4g). We further observed that, similar to BC, *MIR888* hypomethylation also occurs in all cancer types with reported miR-888-5p overexpression, which arise from tissues other than testis, such as prostate [32, 33], endometrial [34], breast [35], hepatocellular [36], and colon tumors [37] (Additional File 4, Figure S3). Thus, we speculated that miR-888-5p levels across cancers may commonly increase as a consequence of gene hypomethylation.

In support of a hypomethylation-induced activation of *MIR888*, we predicted a CpG island upstream of the E2F1 binding motifs, spanning the region between − 1611 and − 1337 bps upstream of the TSS (Fig. 4h, top). Methylation-specific PCR revealed that this region is methylated in non-invasive BC cells, while in high miR-888-5p-expressing, invasive cell lines elevated fractions of unmethylated CpG islands were detected (Fig. 4h, bottom). Moreover, treatment of RT-4 with 5-aza-2-deoxycytidine induced demethylation of the *MIR888* CpG island as opposed to DMSO-treated control cells (Fig. 4i, top), followed by marked miR-888-5p elevation (Fig. 4i, bottom). Hypermethylation of oncogene promoters restricts their expression, but, once deregulated, specific transcription factor(s) have the opportunity to approach these promoters, thereby enhancing gene transcription [43]. In agreement, our data suggest that hypomethylation renders the *MIR888* promoter more accessible to its transcription factors. This event, in combination with E2F1 abundancy in invasive BC cells, facilitates E2F1-mediated upregulation of miR-888-5p, which subsequently acts as an APLF inhibitor.

### E2F1/miR-888-5p/APLF rewiring suppresses c-NHEJ and increases cell invasiveness

**Fig. 5:**
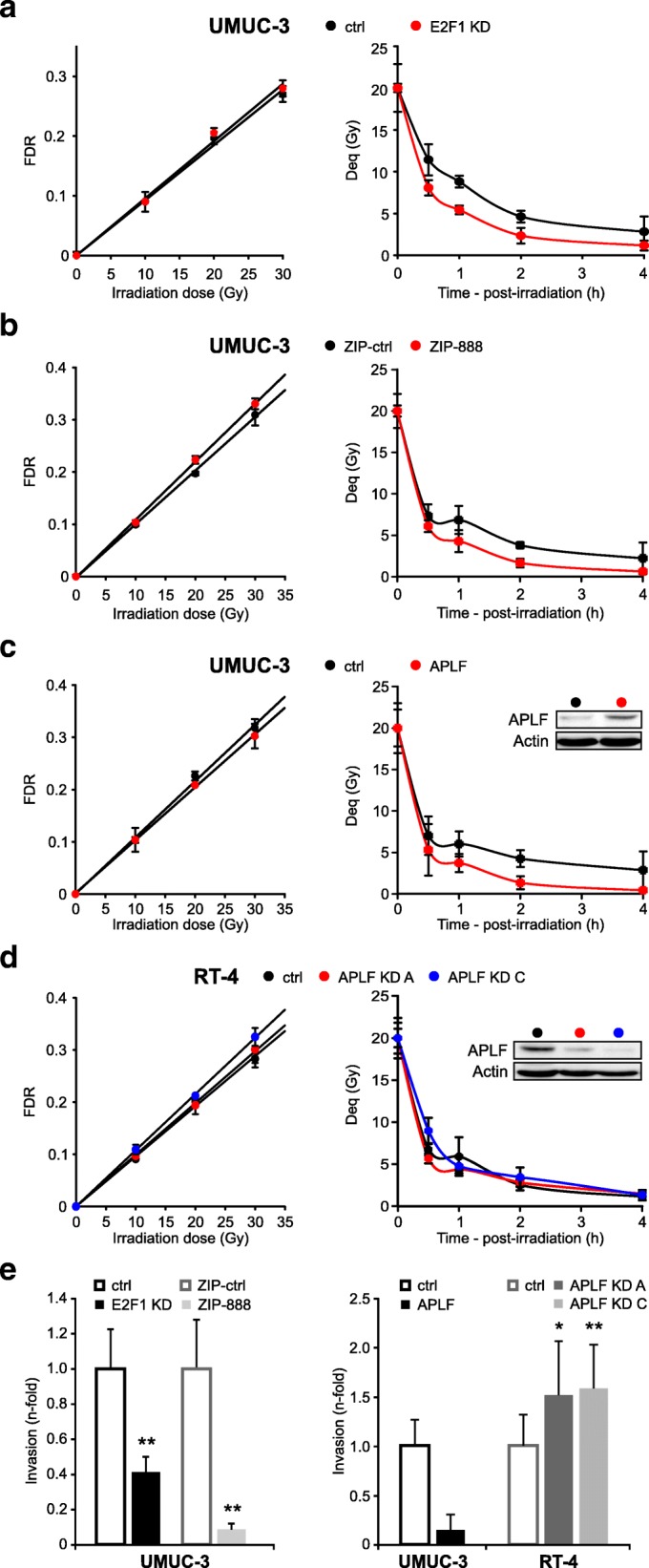
E2F1/miR-888/APLF rewiring impairs c-NHEJ and enhances cell invasiveness. **a-d** PFGE assays estimating the dose-response (*left*) and kinetics of rejoining of DSBs (*right*) in (a) UMUC-3 with E2F1 knockdown (KD), (b) miR-888-5p knockdown (ZIP-888), (c) APLF overexpression, and in (d) RT-4 with APLF knockdown (KD, clones A and C) versus their controls. All PFGE results represent the mean and standard deviation calculated from six determinations in two experiments. APLF protein levels were verified by immunoblotting, using actin as loading control. Impairment of DSB repair due to APLF reduction is evidenced only in aggressive UMUC-3 BC cells with an active E2F1/miR-888-5p pathway, while APLF loss in the less-invasive RT-4 cells in the absence of miR-888-5p has an insignificant effect on DSB repair. **e** Invasion assays of UMUC-3 with E2F1 KD, miR-888-5p KD (*left*) or ectopic APLF expression versus APLF-depleted RT-4 cells (*right*) compared to the controls (* *p* < 0.05, ** *p* < 0.01)

E2F1 upregulates most c-NHEJ factors, but concomitantly downregulates APLF via miR-888 transactivation. The established E2F1/miR-888 axis overall disturbs the relative ratios of the constituents of the c-NHEJ complex, by creating conditions of APLF scarcity in presence of overexpression of the core c-NHEJ factors. We therefore estimated if the E2F1/miR-888-5p/APLF rewiring affects DSB repair and invasion capacity of BC cells. PFGE assays indicated that E2F1 knockdown in UMUC-3 cells enhances DSB repair (Fig. 5a, Additional File 5, Figure S4a). Similarly, a lower amount of unrepaired DSBs was detected in UMUC-3.ZIP-888 cells compared to control (Fig. 5b, Additional File 5, Figure S4b), suggesting improved c-NHEJ activity upon APLF restoration. In an analogous manner, UMUC-3 cells stably expressing APLF (UMUC-3.APLF) showed an increased DSB repair versus control (Fig. 5c, Additional File 5, Figure S4c). Intriguingly, APLF knockdown in less-invasive cells, which bear low endogenous levels of E2F1 and E2F1-upregulated c-NHEJ factors, caused a minor, insignificant impairment of DSB repair (Fig. 5d, Additional File 5, Figure S4d). This suggests that in high E2F1-expressing cells, DSB repair is suppressed by reduced APLF levels and may not benefit from the endogenously increased expression of core c-NHEJ components. The inhibition of DSB repair is achieved through activation of the entire E2F1/miR-888/APLF axis rather than through alterations of APLF levels alone.

We then investigated whether E2F1/miR-888/APLF rewiring favors invasiveness. To this end, we performed invasion assays to test if manipulation of each of the nodes of this regulatory axis influences BC progression. The data shown in Fig. 5e clearly revealed that ablation of either E2F1 or miR-888 (left) as well as APLF overexpression in UMUC-3 cells severely decreases invasive capacity. Vice versa, invasiveness is significantly increased in APLF-depleted RT-4 cells (right). Collectively, these results demonstrate that E2F1/miR-888/APLF rewiring in aggressive BC underlies both reduction of DSB repair and increase of invasive capacity.

### MIR888 knockdown establishes a favorable, bladder cancer-specific E2F1/APLF/DCLRE1C signature

**Fig. 6:**
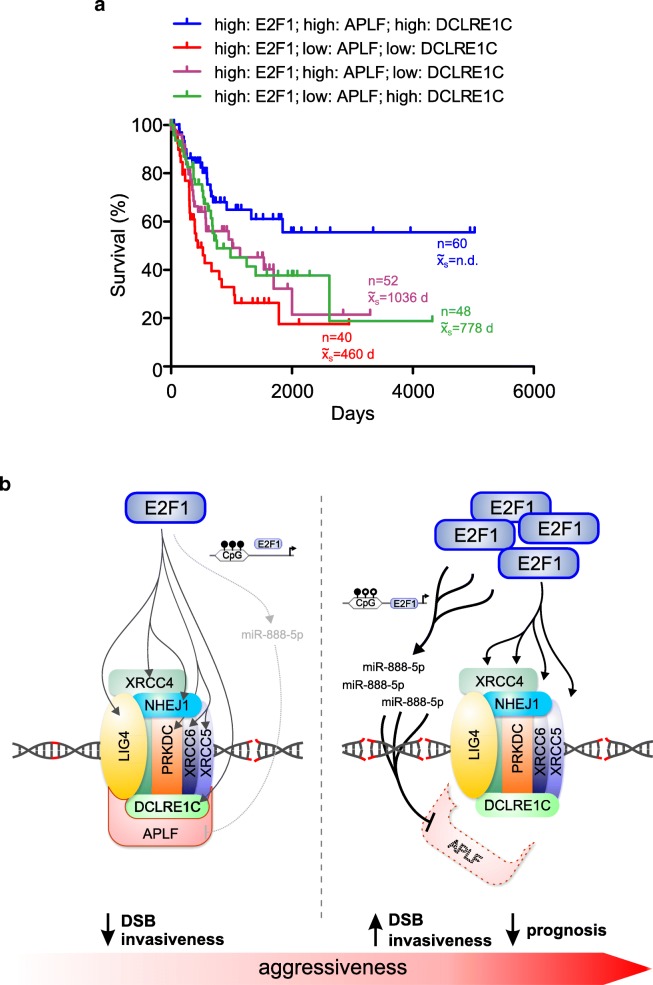
Relative abundances between APLF and c-NHEJ factors in response to E2F1 determine patient survival. **a** OS of BC patients with high E2F1 accompanied by a ‘high APLF/high DCLRE1C’ (blue), ‘low APLF/low DCLRE1C’ (red), ‘high APLF/low DCLRE1C’ (purple) or ‘low APLF/high DCLRE1C’ (green) tumor signature. Patients with high expression of either APLF (‘high APLF/low DCLRE1C’, purple) or DCLRE1C (‘low APLF/high DCLRE1C’, green) have a longer median survival than patients whose tumors lack APLF plus DCLRE1C (red), but their survival time is shorter than with high expression of both (blue), indicating that these two c-NHEJ factors exert a combinatorial effect on survival of high E2F1-expressing BC patients. **b** Proposed mechanism of the impact of E2F1 on c-NHEJ factors. E2F1 upregulates core factors of c-NHEJ except for APLF. During BC progression, the *MIR888* promoter becomes hypomethylated, a fact that permits its ectopic activation by high levels of E2F1 and results to APLF scarcity. The disturbance in the stoichiometry between APLF and core factors of the c-NHEJ multiprotein complex reduces DSB repair and facilitates aggressiveness

Thus far, our results demonstrate that *MIR888* knockdown in MIBCs improves DSB repair and reduces invasiveness by specifically rescuing APLF levels, while leaving E2F1 and E2F1-regulated c-NHEJ factors essentially elevated. This anti-invasive effect is not achieved through reversion in a ‘low E2F1/high APLF/low c-NHEJ factor’ expression pattern, which, as shown in Fig. 1b and c, characterizes NMIBC cells, but rather by establishing a novel condition of ‘high E2F1/APLF/c-NHEJ factor’. To evaluate if this condition is clinically meaningful for survival, we checked whether TCGA BC patients with a ‘high E2F1/APLF’ expression profile exhibit improved OS, when each one of the E2F1-regulated NHEJ factors also remains high. Kaplan-Meier curves showed that these patients do not benefit from co-elevation of LIG4, NHEJ1, PRKDC, XRCC4, XRCC5 or XRCC6 (Additional File 6, Figure S5). Patients do however benefit from co-elevation of DCLRE1C (Fig. 6a, blue line). Interestingly, we further noticed that combined low expression of DCLRE1C and APLF correlates with worse OS (Fig. 6a, red line). Moreover, the OS for patients with high E2F1 and increased level of either APLF or DCLRE1C (Fig. 6a, purple and green line, correspondingly) is better than survival for patients with a ‘low APLF/DCLRE1C’ profile, but nonetheless does not exceed the OS of the patients highly expressing both proteins. Hence, in a high E2F1 context, presence of at least one c-NHEJ factor with nuclease activity [8], either APLF or DCLRE1C, can partially rescue OS versus patients with insufficiency in both nucleases. Correlations between E2F1/APLF and miR-888 were not performed due to inadequate miR-888 expression data in the current TCGA patient cohorts. Overall, our results suggest that APLF and DCLRE1C exert a combinatorial effect on OS, enabling E2F1 to retain a bona fide character and that this particularly favorable ‘high E2F1/APLF/DCLRE1C’ signature can be established by depletion of miR-888-5p.

Given that E2F1 overexpression correlates with poor patient outcomes in several cancer types [29], we performed further Kaplan-Meier analyses in the PAN Cancer cohort to check whether this combination of high DCLRE1C and APLF mRNA levels discriminates between better and worse OS in high E2F1 tumors, in additional cancer types. We found that the ‘high E2F1/APLF/DCLRE1C’ signature does not produce significantly different outcomes across multiple cancer types (Additional File 7, Figure S6), but instead it is highly specific for BC.

## Discussion

Herein, we show for the first time, that E2F1 controls c-NHEJ as a whole via direct transactivation of the majority of c-NHEJ factors, including DCLRE1C, LIG4, NHEJ1, PRKDC, XRCC4, XRCC5, and XRCC6. In parallel, E2F1 promotes miR-888-5p expression as a consequence of *MIR888* hypomethylation, which in turn downregulates APLF in MIBCs. This leads to impairment of DSB repair and is accompanied by enhanced cell invasion. The effect is highly dependent on the E2F1/miR-888 context.

Collectively, our data underscore that a dysfunctional c-NHEJ machinery is attributed to disturbance of the relative ratios between APLF and several c-NHEJ factors in response to high E2F1 (Fig. 6b). For efficient repair, adequate APLF levels should exist to interact with Ku80 and XRCC4-LIG4 [9]. The excess of the E2F1-transactivated c-NHEJ factors plausibly creates an increased demand for APLF at the DSB sites. For instance, increased numbers of Ku molecules require more Ku Binding Domains of APLF [44] as anchor points for triggering the c-NHEJ network reactions. However, since E2F1/*MIR888* activation jeopardizes APLF abundancy, E2F1 overexpression ultimately creates conditions of APLF scarcity for the multiprotein complex assembly. This APLF shortage worsens DSB repair in the face of high expression of core c-NHEJ factors. Contrary to other c-NHEJ factors, APLF is not a direct E2F1 target and therefore, it cannot increase proportionally to them in a high E2F1 cell context. The detrimental effect of E2F1 on c-NHEJ functionality relies on the fact that the hypomethylated E2F1-responsive miR-888-5p suppresses a critical c-NHEJ component that cannot be replenished in high E2F1-expressing cells. This plausibly establishes an unbalanced stoichiometry between APLF and other components of the c-NHEJ complex. Under these conditions, excessive levels of the E2F1-regulated c-NHEJ factors do not compensate for APLF insufficiency in terms of c-NHEJ repair, cell invasiveness, and ultimately, BC patient survival.

Since the c-NHEJ factors act as a multiprotein complex, we further propose that changes in the relative ratios of several constituents of the complex might be more representative indicators of patient outcomes, as compared to changes of the absolute levels of individual c-NHEJ factors. This would explain why, in some BC patient cases, the individually high levels of e.g. Ku70 or Ku80 fail to correlate [7] or are even negatively correlated with survival, notwithstanding that their abundance is, at first glance, indicative of a functional c-NHEJ [8]. Here, we show that combinations of factors comprising the c-NHEJ repair complex, together with their major upstream c-NHEJ transcriptional regulators could provide appropriate and BC-specific diagnostic signatures. In particular, we unveiled that survival is specifically correlated with high levels of the nucleases APLF and DCLRE1C, which exert the ability to process incompatible single-stranded overhangs to create blunt ends that can be ligated. DCLRE1C is the main nuclease, while APLF may participate, especially when DCLRE1C is not present. End-joining cannot be performed unless DCLRE1C is present and its function cannot be surrogated by the presence of other factors such as Ku, DNA-PKcs and LIG4 [45]. Impaired c-NHEJ is associated with more invasive phenotypes and this should be reflected accordingly in patient survival. In this regards, the existence of a favorable ‘high E2F1/APLF/DCLRE1C’ signature could possibly underscore the presence of a still functional DSB repair machinery which sets a barrier on invasive tumor growth, thereby keeping E2F1 at the ‘bright side’ of DNA repair. E2F1 passes to the ‘dark side’ [18] and signifies worse outcomes only after rewiring with the hypomethylated miR-888, which distorts the ratio between APLF and other components of the c-NHEJ complex. Selective miR-888-5p knockdown establishes a ‘high E2F1/APLF/DCLRE1C’ signature, which is associated with reduced invasiveness and improved survival.

Furthermore, perturbation of the E2F1/miR-888/APLF axis increases DSB repair and reduces invasiveness, suggesting that this pathway commonly underlies both processes (Fig. 6b). Theoretically, an association between c-NHEJ deficiency and acquisition of invasive properties via common underlying pathways could be inferred, considering that tumors follow evolutionary principles. Since c-NHEJ-impaired BC cells resort to alt-EJ [2, 7], they become more prone to mutagenic DSB repair and genomic instability [3, 46]. Genomic instability can greatly increase intratumoral cell diversity and accelerate somatic evolution [47], because a tumor consisting of a genetically heterogeneous cell population has more possibilities to respond to microenvironmental changes, to evolve, and to spread [48, 49]. In this respect, it would be interesting to use single-cell sequencing approaches to track the evolutionary trajectory of a BC tumor and unveil whether E2F1-triggered c-NHEJ dysregulation might consist one of the mechanisms orchestrating the evolutionary adaptation towards aggressiveness and resistance to therapy.

*MIR888* is a testis-restricted, higher primate-specific, X-linked miRNA gene, important for sperm motility and male fertility [38]. In many normal tissues it remains dormant, but it often gets transactivated in tissues other than testis, and acts as an oncogene (ref. [32] and this study), if its hypermethylation-mediated silencing is bypassed. The consistent ability of miR-888 to orchestrate cancer invasiveness [32] when expressed in a naïve tissue environment might be due to the fact that non-testis tissues could inherently lack miR-888-inhibiting factors and/or negative feedback loops to counteract miR-888-5p overexpression. Nevertheless, the disadvantage of miR-888-5p expression in a new tissue environment can be turned into a therapeutic advantage. This is because a drug inactivating *MIR888* is anticipated to be highly selective for BC cells and may cause less side-effects and/or toxicity in adjacent healthy tissues, which do not normally express this miRNA.

APLF restoration via miR-888 targeting improves DSB repair, prevents invasive outcomes, and could be therapeutically beneficial for high E2F1-expressing BC patients. Still, the role of APLF in cancer progression remains controversial and could be cancer type- and/or cell context-specific. In support of this notion, APLF activity in BC depends on the miR-888 context, whereas in a triple-negative breast cancer cell line with no miR-888-5p expression [34, 38] excessive APLF triggers epithelial-to-mesenchymal transition (EMT) [50, 51]. The effect of APLF on EMT has been attributed to its histone chaperone activity [50, 51]. Given that APLF encompasses various functional domains through which it acts both as a chaperone and an endonuclease, its effect on EMT could be occurring independently from its protein-protein interactions with other core factors of c-NHEJ. A more intriguing hypothesis is that these functional surfaces could serve as a comprehensive platform that co-ordinates EMT in conjunction to c-NHEJ, depending on the relative abundances of its interacting core c-NHEJ factors. These questions remain a subject of future research.

## Conclusion

We unveil the mechanism of the E2F1-dependent impairment of c-NHEJ machinery and show its impact on DSB repair and bladder cancer progression. E2F1 directly transactivates c-NHEJ core factors, but in parallel, it accesses the hypomethylated promoter of *MIR888* and upregulates miR-888, an APLF inhibitor, in a tissue where it is normally epigenetically silent. This overall disturbs the relative ratios of the core factors and creates conditions of APLF scarcity. Suppression of the ectopically activated miR-888 restores APLF, improves DSB repair and impedes invasiveness. Thus, the pharmacological suppression of the ‘out-of-context’ activity of this miRNA may hold promise for efficacy against E2F1-positive invasive bladder cancer and raises new hope in the miRNA therapeutics field.

## Additional files

Additional file 1: Supplemental materials and methods. **Table S1.** Detection primers used in semi-quantitative PCR and quantitative PCR. **Table S2.** Primers for E2F1 binding sites used in ChIP assay. **Table S3.** Raw and Bonferroni-adjusted *p* values for the Kaplan-Meier plots of the indicated figures. (DOCX 39 kb)

Additional file 2: **Figure S1.** Quantification of mRNA levels in UMUC-3.ZIP.888. (PDF 1611 kb)

Additional file 3: **Figure S2.** Expression levels of miR-888-5p across several normal tissues. (PDF 1064 kb)

Additional file 4: **Figure S3.** Methylation status of the *MIR888* promoter. (PDF 2364 kb)

Additional file 5: **Figure S4.** Representative agarose gels demonstrating the fraction of DNA released (FDR) from the well into the gel for UMUC-3 E2F1 KD, miR-888-5p KD, APLF overexpression, and RT-4 APLF KD. (PDF 35 kb)

Additional file 6: **Figure S5.** OS of BC patients with a high E2F1/high APLF tumor content upon elevated mRNA expression of the c-NHEJ complex factors. (PDF 2143 kb)

Additional file7: **Figure S6.** OS of PAN Cancer cohort patients with a ‘high E2F1/high APLF/high DCLRE1C’ tumor signature. (PDF 117 kb)

## Data Availability

Supplementary information is available as Additional data files.

## Abbreviations

altEJ: Alternative end joining
BC: Bladder cancer
ChIP: Chromatin immunoprecipitation
c-NHEJ: Classical non-homologous end-joining
DSB: Double strand break
EMT: Epithelial-to-mesenchymal transition
MIBC: Muscle-invasive BC
miRNA: MicroRNA
NMIBC: Non-muscle-invasive bladder carcinoma
OS: Overall survival
PFGE: Pulse field gel electrophoresis
qPCR: Quantitative Real-Time PCR
RT-PCR: Reverse-transcriptase PCR
TSS: Transcription start site
