# Supplementary material for: Rewiring E2F1 with classical NHEJ via APLF suppression promotes bladder cancer invasiveness
Source: J Exp Clin Cancer Res. 2019 Jul 8;38:292. doi: 10.1186/s13046-019-1286-9 (PMC6615232; doi:10.1186/s13046-019-1286-9)
Supplement: Supplementary file 1 — Supplemental materials and methods. Table S1. Detection primers used in semi-quantitative PCR and quantitative PCR. Table S2. Primers for E2F1 binding sites used in ChIP assay. Table S3. Raw and Bonferroni-adjusted p values for the Kaplan-Meier plots of the indicated figures. (DOCX 39 kb) [file 13046_2019_1286_MOESM1_ESM.docx]

**Rewiring E2F1 with classical NHEJ via APLF suppression promotes bladder cancer invasiveness**

**Christin Richter^1^, Stephan Marquardt^1^, Fanghua Li^2^, Alf Spitschak^1^, Nico Murr^1^, Berdien AH Edelhäuser^1^, George Iliakis^2^, Brigitte M Pützer^1,3,†,*^, Stella Logotheti^1,†^**

^1^Institute of Experimental Gene Therapy and Cancer Research, Rostock University Medical Center, Rostock, Germany

^2^Institute of Medical Radiation Biology, University of Duisburg-Essen Medical School, Essen, Germany

^3^Department Life, Light and Matter of the Interdisciplinary Faculty at Rostock University, Rostock, Germany

E-mail addresses:

Christin Richter: christin.richter@med.uni-rostock.de

Stephan Marquardt: stephan.marquardt@med.uni-rostock.de

Fanghua Li: Fanghua.Li@uk-essen.de

Alf Spitschak: alf.spitschak@med.uni-rostock.de

Nico Murr: nico.murr@uni-rostock.de

Berdien AH Edelhäuser: berdien.jansen@uni-rostock.de

George Iliakis: Georg.Iliakis@uk-essen.de

Brigitte M Pützer: brigitte.puetzer@med.uni-rostock.de

Stella Logotheti: Styliani.Logotheti@med.uni-rostock.de

*Correspondence to: Brigitte M. Pützer, Institute of Experimental Gene Therapy and Cancer Research, Rostock University Medical Center, Schillingallee 69, 18057 Rostock, Germany. Phone: +49 (0)381 494-5066/68; Fax: +49 (0)381 494-5062; brigitte.puetzer@med.uni-rostock.de

†Equally contributing senior authors.

**Supplementary materials and methods**

**Cell culture and treatments**

RT-4, UMUC-3 and T24 cell lines were purchased from ATCC (Rockville, MD, USA) and kindly provided by Dr S. Füssel, Urology Laboratory, University of Dresden. UMUC-3 and T24 cells were maintained at 37 °C and 5% CO2 in Dulbecco’s modified Eagle’s medium (high glucose, 4.5 g/l) containing 2 mM L-glutamine, 1 mM sodium pyruvate, supplemented with 10% FCS, 0.1 mM non-essential amino acids, 50 U/ml Penicillin and 50 µg/ml Streptomycin. RT-4 cells were grown in RPMI medium with the same supplements. All cell lines were authenticated and also tested for mycoplasma contamination prior to the experiments according to manufacturer’s instructions (Venor GeM Classic, Minerva Biolabs).

For methylation analysis of the *MIR888* promoter, 3x10^6^ RT-4 cells were plated in 10-cm cell culture dishes and treated with the demethylating agent 5-aza-deoxycytidine (AZA, from a 50μg/μL stock dilution in DMSO) at a final concentration of 1μM. Fresh AZA-containing medium was added every 24 h for 5 days. DMSO-treated cells were used as negative controls. Cells were pelleted and subjected to miRNA and genomic DNA isolation.

**Genomic DNA isolation, bisulfite treatment, and methylation-specific PCR**

Genomic DNA was extracted from RT-4, T24, UMUC-3, and RT-4-AZA treated cells using the DNeasy kit (Qiagen, Hilden, Germany) according to the manufacturer’s protocol. For methylation analysis, the UCSC Genome Browser on Human Dec. 2013 (GRCh38/hg38) Assembly was used to scan for masked and unmasked CpG islands in a chromosomal region spanning 2,000 base pairs (bps) upstream of *MIR888*. Primers were designed for methylation-specific PCR, enhancing the methylated or the unmethylated version of the predicted CpG island using the MethPrimer2 tool [1]. Methylated CpG island primers were: Fwd: 5’-AGAAGAATAGAAAATGGAATAAGATGGTC-3’, Rev: 5’-TAAAACGAAATCTCGCTCTATCGCC-3’. Unmethylated CpG island primers were: Fwd: 5’-AAGAATAGAAAATGGAATAAGATGGTT-3’, Rev: 5’-TTTAAAACAAAATCTCACTCTATCACCC-3’. Overall, 500 ng of genomic DNA were treated with sodium bisulfite using the EZ DNA methylation Kit™ (ZymoResearch, CA, USA) and diluted in 10 μl according to the instructions. Four μl were used in each PCR reaction either with the unmethylated or methylated primer pair. The universal methylated human DNA standard (ZymoResearch, CA, USA) was used as control for *MIR888* CpG methylation.

**Plasmid construction and transfection**

The pcDNA3.1-E2F1 expression plasmid has been described earlier [2]. For the pcDNA3.1-miR-888 expression plasmid, a 595 bp DNA fragment containing the *MIR888* gene was PCR-amplified from genomic DNA of T24 cells with the following primers: Fwd: 5’-TCTTGCCTCGAGCTGGTCCTGGCAATCATCCAGATA-3’ and Rev: 5‘-GAATTTACGCGTCACTGCCCTCCAACTTGGCAT-3’ and cloned into pcDNA3.1(+) vector using the TopoTA cloning kit (Thermo Fisher Scientific). The pMIR-REPORT-3 APLF luciferase construct (3´UTRAPLF-Luc) was generated by amplification of a 314 bp fragment of the 3´UTR of the APLF gene containing two predicted miR-888-5p binding sites using the Fwd: 5‘-TAGCTACTAGTGGATTTCCTTTTGAGTGATAAA-3‘ and Rev: 5‘-TAGCTAAGCTTTAGTTATACTCAAAAGTGTCTTCAGG-3‘ primers and Phusion® High-Fidelity DNA Polymerase (New England Biolabs). The amplimer was digested with HindIII/SpeI, gel-purified, and cloned into corresponding restriction sites of pMIR-REPORT (Ambion, Applied Biosystems). For the pGL4.10[luc2]-MIR888 promoter luciferase construct (MIR888prom_Luc), a region 611-974 bp upstream of the *MIR888* transcriptional start site (TSS) with the predicted E2F1 binding sites was amplified using Fwd: 5‘-GGTACCTGAGTTTGGCTGGCCTTTGGAC-3‘ and Rev: 5‘-GCCGGAAGCTTACCATGGCGCCTGAACCC-3‘ primers and cloned into pGL4.10[luc2] vector (Promega). All constructs were verified by sequencing analysis (Sequence Laboratories Göttingen GmbH). Transient transfection was performed using TurboFect^TM^ (Thermo Scientific).

**Viral vectors and transduction**

Adenoviral vectors expressing shRNA against E2F1 or scrambled control shRNA were described elsewhere [2]. Transduction of T24 and UMUC-3 was performed at MOI 15 for 72 h. The miRZIP-888 anti-miR-888 microRNA lentiviral vector (MZIP888-PA-1) was purchased from System Biosciences. APLF-set siRNA Lentivector (i001153) expressing siAPLF RNA and pLenti-APLF-Puro2AGFP vector expressing APLF mRNA (LV077673) were purchased from Applied Biological Materials. The pLenti-APLF-Puro2AGFP vector was further modified by double digest with PmeI and BmtI followed by by Klenow treatment and re-ligation to achieve in-frame expression of APLF protein. VSV-G-enveloped pseudotyped lentiviral vectors were generated by co-transfecting HEK 293T cells with the expression plasmid and both packaging plasmids, psPAX2 and pMD2.G, from Addgene as described previously [3]. Stable clones like RT-4 expressing shRNA against APLF (RT-4 APLF KD A or C), UMUC-3.APLF, and T24 or UMUC-3 expressing miR-888-5p antagomiR (T24.ZIP-888 and UMUC-3.ZIP-888) and their controls were generated under puromycin selection (2 mg/ml).

**Generation of E2F1 knockdown cells by CRISPR-Cas9**

Plasmid pLKO5.sgRNA.Cas9.EFS.eGFP was used to stably express Cas9 in UMUC-3. Sequences of the guide RNA for E2F1 knockdown were designed *in silico*: sgE2F1-fwd: 5’- CACCGCGTCATCATCTCCGCCGCGC-3’; sgE2F1-rev: 5’- AAACGCGCGGCGGAGATGATGACGC-3’. After annealing and phosphorylation, the oligos were cloned into the BsmBI site of pLKO5.sgRNA.EFS.PAC (obtained from D. Heckl). Following lentiviral vector production and transduction, single cell clones expressing guide RNAs were selected with puromycin (2 mg/ml). Stable UMUC-3.CRISPR-Cas9-sgE2F1 knockdown cells and the UMUC-3.Cas9 control clones were produced, and E2F1 and Cas9 expression was validated by immunoblots with anti-E2F1 (#3742, Cell Signaling) and anti-Cas9 (bd-20, sc-392737, Santa Cruz) antibodies.

**Chromatin immunoprecipitation**

ChIP was performed as described [2]. Cell lysates were incubated overnight with or without anti-E2F1 followed by incubation for 1 h with Protein A/G Plus-Agarose (sc-2003, Santa Cruz). The immunoprecipitated DNA fragments were amplified by PCR or qPCR. The values were normalized by the fold-enrichment method, whereby the ChIP signal of each E2F1-antibody-treated sample was divided by the signal of corresponding mock IP, which represents the background signal [4]. The result is represented as the fold increase in signal in E2F1 antibody-treated samples relative to the background signal [5]. The E2F1 binding site of *APAF-1* promoter [6] was used as a positive control for functional E2F1 binding sites. Primers for binding sites are listed in Table S2. Ten percent input sample, representing the amount of chromatin used in ChIP, was used as a control of PCR enhancement for each primer pair.

**Luciferase reporter assays**

RT-4 cells were transiently co-transfected with 1 µg pcDNA3.1-miR-888 or pcDNA3.1 and 1 µg 3´UTRAPLF-Luc plasmid. T24.ZIP-888 or UMUC-3.ZIP-888 cells and their controls were transiently transfected with 1 µg 3´UTRAPLF-Luc plasmid. Reporter activity was measured 24 h after transfection using the Luciferase Reporter Assay System (Promega). Samples were normalized to total protein concentration in cell extracts. UMUC-3 E2F1 knockdown cells or their scrambled shRNA-expressing controls were transiently co-transfected with 1,5 µg MIR888prom_Luc plasmid and 1,5 µg pGL4.75 [hRLuc/CMV] and activity was measured 24 h later using the Dual-Luciferase Reporter Assay System (Promega).

**Bioinformatics analysis**

Sequences were downloaded from NCBI. E2F1 binding sites were predicted by Pscan software [7] using the Refseq mRNA transcript IDs, PWMs from JASPAR (2016), range of -950/+50 bp relative to TSS and the “occurrence” function. We also used JASPAR software to screen promoters of up to 5 kb length. ConTra v3 software [8] was used to check binding site conservation in the 5’UTR of each gene. MiRNAs that target APLF were predicted using the DIANA-microT-CDS platform [9]. The *MIR888* expression profiles across human tissues were mined from the miRIAD database [10]. The DiseaseMeth version 2.0 [11] was used to estimate *MIR888* methylation status in several cancer types with the following analysis options: array and NGS-based technology Experimental Platforms, controls of the same tissue/cell-line, t-test method of differential analysis, p-value of 0.05, and absolute methylation difference > 0.2. Patient RNA-Seq data from TCGA cohorts with bladder cancer and from the PAN Cancer cohort were retrieved from Xena browser (<https://xenabrowser.net/>). High (above defined threshold) or low (below defined threshold) expression of E2F1 and c-NHEJ factors (APLF, DCLRE1C, LIG4, PRKDC, XRCC4, XRCC5, and XRCC6) was defined by setting a threshold T for E2F1 T = 9.2 (median = 9.124), for APLF T = 6.0 (median = 6.22), for all other genes T = median and patients were grouped according to the combined expression pattern of high E2F1 and the c-NHEJ factors as shown in the respective figures. Overall-survival (OS) and recurrence-free survival (RFS) curves were analyzed by the Kaplan-Meier method. Data were plotted using GraphPad Prism. The pairwise p values presented in Table S3 were obtained from log-rank (Mantel-Cox) tests in GraphPad Prism and corrected for multiple-hypothesis testing using Bonferroni correction with n = 8 in the analysis for Fig. 3a, b, n = 3 in the analysis for Fig. 6a, and n = 6 in the analysis for Fig. S5.

**References**

1. Li LC, Dahiya R (2002) MethPrimer: designing primers for methylation PCRs. Bioinformatics 18 (11):1427-1431

2. Alla V, Engelmann D, Niemetz A, Pahnke J, Schmidt A, Kunz M, Emmrich S, Steder M, Koczan D, Putzer BM (2010) E2F1 in melanoma progression and metastasis. J Natl Cancer Inst 102 (2):127-133. doi:10.1093/jnci/djp458

3. Salmon P, Trono D (2007) Production and titration of lentiviral vectors. Curr Protoc Hum Genet Chapter 12:Unit 12.10. doi:10.1002/0471142905.hg1210s54

4. Nelson JD, Denisenko O, Bomsztyk K (2006) Protocol for the fast chromatin immunoprecipitation (ChIP) method. Nat Protoc 1 (1):179-185. doi:10.1038/nprot.2006.27

5. Galtsidis S, Logotheti S, Pavlopoulou A, Zampetidis CP, Papachristopoulou G, Scorilas A, Vojtesek B, Gorgoulis V, Zoumpourlis V (2017) Unravelling a p73-regulated network: The role of a novel p73-dependent target, MIR3158, in cancer cell migration and invasiveness. Cancer Lett 388:96-106. doi:10.1016/j.canlet.2016.11.036

6. Moroni MC, Hickman ES, Lazzerini Denchi E, Caprara G, Colli E, Cecconi F, Müller H, Helin K (2001) Apaf-1 is a transcriptional target for E2F and p53. Nat Cell Biol 3 (6):552-558. doi:10.1038/35078527

7. Zambelli F, Pesole G, Pavesi G (2009) Pscan: finding over-represented transcription factor binding site motifs in sequences from co-regulated or co-expressed genes. Nucleic Acids Res 37 (Web Server issue):W247-252. doi:10.1093/nar/gkp464

8. Kreft L, Soete A, Hulpiau P, Botzki A, Saeys Y, De Bleser P (2017) ConTra v3: a tool to identify transcription factor binding sites across species, update 2017. Nucleic Acids Res 45 (W1):W490-W494. doi:10.1093/nar/gkx376

9. Maragkakis M, Reczko M, Simossis VA, Alexiou P, Papadopoulos GL, Dalamagas T, Giannopoulos G, Goumas G, Koukis E, Kourtis K, Vergoulis T, Koziris N, Sellis T, Tsanakas P, Hatzigeorgiou AG (2009) DIANA-microT web server: elucidating microRNA functions through target prediction. Nucleic Acids Res 37 (Web Server issue):W273-276. doi:10.1093/nar/gkp292

10. Hinske LC, Franca GS, Torres HA, Ohara DT, Lopes-Ramos CM, Heyn J, Reis LF, Ohno-Machado L, Kreth S, Galante PA (2014) miRIAD-integrating microRNA inter- and intragenic data. Database (Oxford) 2014. doi:10.1093/database/bau099

11. Xiong Y, Wei Y, Gu Y, Zhang S, Lyu J, Zhang B, Chen C, Zhu J, Wang Y, Liu H, Zhang Y (2017) DiseaseMeth version 2.0: a major expansion and update of the human disease methylation database. Nucleic Acids Res 45 (D1):D888-D895. doi:10.1093/nar/gkw1123

| primer name | sequence (5´🡪3´) |
| --- | --- |
| Actin_F | CGGGAAATCGTGCGTGACATTA |
| Actin_R | ACCGCTCATTGCCAATGGTGAT |
| APLF_F | TGAGGCAATGAGCTGTTCTG |
| APLF_R | CATGCAGGATGTCCTCTTGA |
| DCLRE1C_F | AAAGAAGGTTGGAGTGCAGCTTG |
| DCLRE1C_R | CCAGCTGGTAAGAGAGTCACAAC |
| GAPDH_F | ATCGTGGAAGGACTCATGACCACA |
| GAPDH_R | AAGGCCATGCCAGTGAGCTTC |
| LIG4_F | CTCAGAGTTCAGCACTTGAGCA |
| LIG4_R | GAGTCCTACAGAAGGATCATGCAG |
| NHEJ1_F | CACTGATTCTACGGGTGCGA |
| NHEJ1_R | AACGTTGCTAGCTCCCTCAC |
| PRKDC_F | CTTTGTCGTGTGGAGGGAAT |
| PRKDC_R | CACAACGGGGTTCAGAAGTT |
| XRCC4_F | TTGTTGTCAGGAGCAGGACC |
| XRCC4_R | TCTGCAATGGTGTCCAAGCA |
| XRCC5_F | GGACGTGGGCTTTACCATGA |
| XRCC5_R | GGGGATTGTCAGTGCCATCT |
| XRCC6_F | CCGAGATACAGGCATCTTCCT |
| XRCC6_R | AGCTTTAACCTGCTGAGTGCT |

**Table S1:** Primers used in semi-quantitative PCR and quantitative PCR

| primer name | sequence (5´🡪3´) |
| --- | --- |
| APAF-1_BS_F | GCCCCGACTTCTTCCGGCTCTTCA |
| APAF-1_BS_R | GGAGCTGGCAGCTGAAAGACTC |
| APLF_BS1_F | CGTCAGCGTTGACTGTCACT |
| APLF_BS1_R | TTCGAATTCCGGTGGATTTA |
| APLF_BS2_F | TAAATCCACCGGAATTCGAA |
| APLF_BS2_R | AGTCCTGGTGGCTTTCTTCA |
| APLF_BS3_F | TGTCTGTGGAGGGCGGAA |
| APLF_BS3_R | GATCACCGTCTCCCCGGG |
| DCLRE1C_BS_F | CCTTTCCTGCCATTTTTCTTT |
| DCLRE1C_BS_R | CTTGTGGGATTACCGTGAGG |
| LIG4_BS_F | CTGTAGGGCGTCACGTTG |
| LIG4_BS_R | CTGCCACCCCACGTAAAC |
| NHEJ1_BS_F | GGGAGAACGTAAATCCATGC |
| NHEJ1_BS_R | GCTCCAGTTCTTCCATCTGC |
| PRKDC_BS_F | CCCTCGCTTGTTTTATCTGC |
| PRKDC_BS_R | ACCCGGAAATGCCCCTAC |
| XRCC4_BS_F | ATCTGGGGGAAAAGAAGTGG |
| XRCC4_BS_R | CAGAGGCCCATAAATGGAAA |
| XRCC5_BS_F | AGCTCTGACACAAAATGCCTTA |
| XRCC5_BS_R | ATGCGCACATTCTCTCCATT |
| XRCC6_BS2_F | GAGATCTCATTGGGCGTGAT |
| XRCC6_BS2_R | CCCAGTAGCTGCCTCATCTC |
| MIR888_BS1_F | AGGGTTCAGGCATTCTGTTG |
| MIR888_BS1_R | TCGAGAAGGCAGTTGAAAGG |
| MIR888_BS2_F | AGTTGAAGGGCAATGAGCTG |
| MIR888_BS2_R | TAATCTGGGCGACTGACACA |

**Table S2:** Primers for E2F1 binding sites used in ChIP assay

| **Fig. 3a, b** | | | |
| --- | --- | --- | --- |
| **patient population** | **versus patient population** | **p value** | **p_adj (n=8)** |
| high E2F1/high DCLRE1C | high E2F1/low DCLRE1C | 0.0049 | 0.0392 |
| high E2F1/high APLF | high E2F1/low APLF | 0.0142 | 0.1136 |
| high E2F1/high LIG4 | high E2F1/low LIG4 | 0.1992 | 1 |
| high E2F1/high NHEJ1 | high E2F1/low NHEJ1 | 0.1102 | 0.8816 |
| high E2F1/high PRKDC | high E2F1/low PRKDC | 0.6856 | 1 |
| high E2F1/high XRCC4 | high E2F1/low XRCC4 | 0.0905 | 0.724 |
| high E2F1/high XRCC5 | high E2F1/low XRCC5 | 0.8041 | 1 |
| high E2F1/high XRCC6 | high E2F1/low XRCC6 | 0.4608 | 1 |
|  | | | |
| **Fig. 3c** | | | |
| high E2F1/high APLF | high E2F1/low APLF | 0.002 | - |
|  | | | |
| **Fig. 6a** | | | |
| **patient population** | **versus patient population** | **p value** | **p_adj (n=3)** |
| high E2F1/high APLF/high DCLRE1C | high E2F1/low APLF/low DCLRE1C | 0.0002 | 0.0006 |
| high E2F1/high APLF/high DCLRE1C | high E2F1/high APLF/low DCLRE1C | 0.0264 | 0.0792 |
| high E2F1/high APLF/high DCLRE1C | high E2F1/low APLF/high DCLRE1C | 0.0526 | 0.1578 |
|  | | | |
| **Fig. S5** | | | |
| **patient population** | **versus patient population** | **p value** | **p_adj (n=6)** |
| high E2F1/high APLF/high NHEJ1 | high E2F1/high APLF/low NHEJ1 | 0.1279 | 0.7674 |
| high E2F1/high APLF/high LIG4 | high E2F1/high APLF/low LIG4 | 0.2411 | 1 |
| high E2F1/high APLF/high PRKDC | high E2F1/high APLF/low PRKDC | 0.6677 | 1 |
| high E2F1/high APLF/high XRCC4 | high E2F1/high APLF/low XRCC4 | 0.8027 | 1 |
| high E2F1/high APLF/high XRCC5 | high E2F1/high APLF/low XRCC5 | 0.5746 | 1 |
| high E2F1/high APLF/high XRCC6 | high E2F1/high APLF/low XRCC6 | 0.882 | 1 |

**Table S3:** Raw and Bonferroni-adjusted p values for the Kaplan-Meier plots of the indicated figures.
