# Supplementary material for: Rewiring E2F1 with classical NHEJ via APLF suppression promotes bladder cancer invasiveness
Source: J Exp Clin Cancer Res. 2019 Jul 8;38:292. doi: 10.1186/s13046-019-1286-9 (PMC6615232; doi:10.1186/s13046-019-1286-9)
Supplement: Supplementary file 2 — Figure S1. Quantification of mRNA levels in UMUC-3.ZIP.888. (PDF 1611 kb) [file 13046_2019_1286_MOESM2_ESM.pdf]

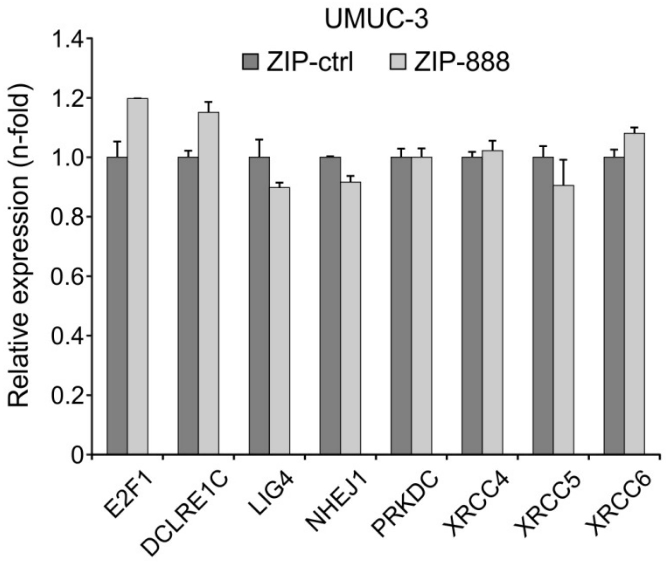

Fig. S1 Quantification of mRNA levels in UMUC-3.ZIP.888. QPCR of E2F1, DCLRE1C, LIG4, PRKDC, XRCC4, XRCC5, XRCC6 in UMUC-3 ZIP-888 clone versus control.
