# Supplementary material for: Rewiring E2F1 with classical NHEJ via APLF suppression promotes bladder cancer invasiveness
Source: J Exp Clin Cancer Res. 2019 Jul 8;38:292. doi: 10.1186/s13046-019-1286-9 (PMC6615232; doi:10.1186/s13046-019-1286-9)
Supplement: Supplementary file 3 — Figure S2. Expression levels of miR-888-5p across several normal tissues. (PDF 1064 kb) [file 13046_2019_1286_MOESM3_ESM.pdf]

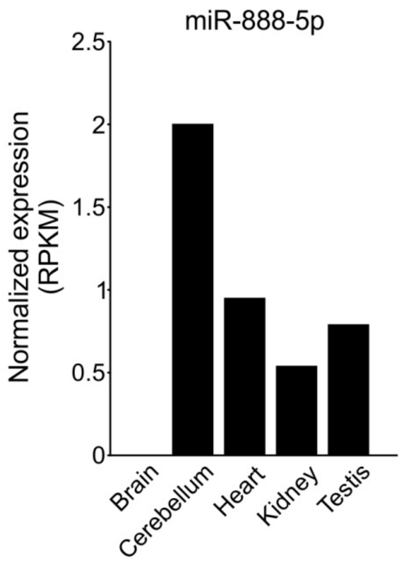

Fig. S2 Expression levels of miR-888-5p across several normal tissues. Data were obtained from MiRIAD database.
