# Supplementary material for: Rewiring E2F1 with classical NHEJ via APLF suppression promotes bladder cancer invasiveness
Source: J Exp Clin Cancer Res. 2019 Jul 8;38:292. doi: 10.1186/s13046-019-1286-9 (PMC6615232; doi:10.1186/s13046-019-1286-9)
Supplement: Supplementary file 4 — Figure S3. Methylation status of the MIR888 promoter. (PDF 2364 kb) [file 13046_2019_1286_MOESM4_ESM.pdf]

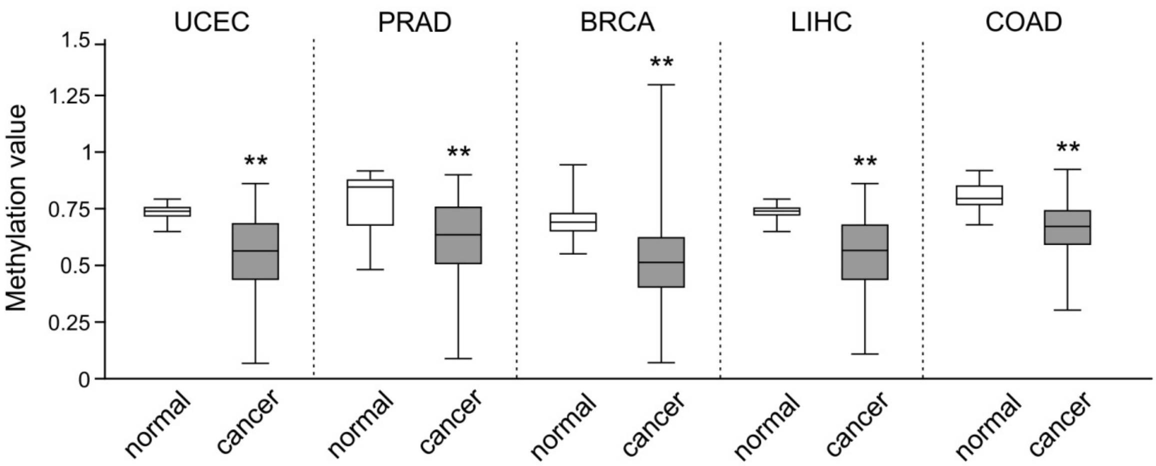

Fig. S3 Methylation status of the MIR888 promoter. Analysis of the promoter region ranging 2000 bps upstream of the transcription start site in tumor types with reported overexpression of miR-888-5p (** p<0.01). UCEC: Uterine Corpus Endometrial Carcinoma, PRAD: Prostate adenocarcinoma, BRCA: Breast Invasive Carcinoma, and LIHC: Liver Hepatocellular Carcinoma. COAD: colon adenocarcinoma. (** *p*<0.01)
