# Supplementary material for: Rewiring E2F1 with classical NHEJ via APLF suppression promotes bladder cancer invasiveness
Source: J Exp Clin Cancer Res. 2019 Jul 8;38:292. doi: 10.1186/s13046-019-1286-9 (PMC6615232; doi:10.1186/s13046-019-1286-9)
Supplement: Supplementary file 5 — Figure S4. Representative agarose gels demonstrating the fraction of DNA released (FDR) from the well into the gel for UMUC-3 E2F1 KD, miR-888-5p KD, APLF overexpression, and RT-4 APLF KD. (PDF 35 kb) [file 13046_2019_1286_MOESM5_ESM.pdf]

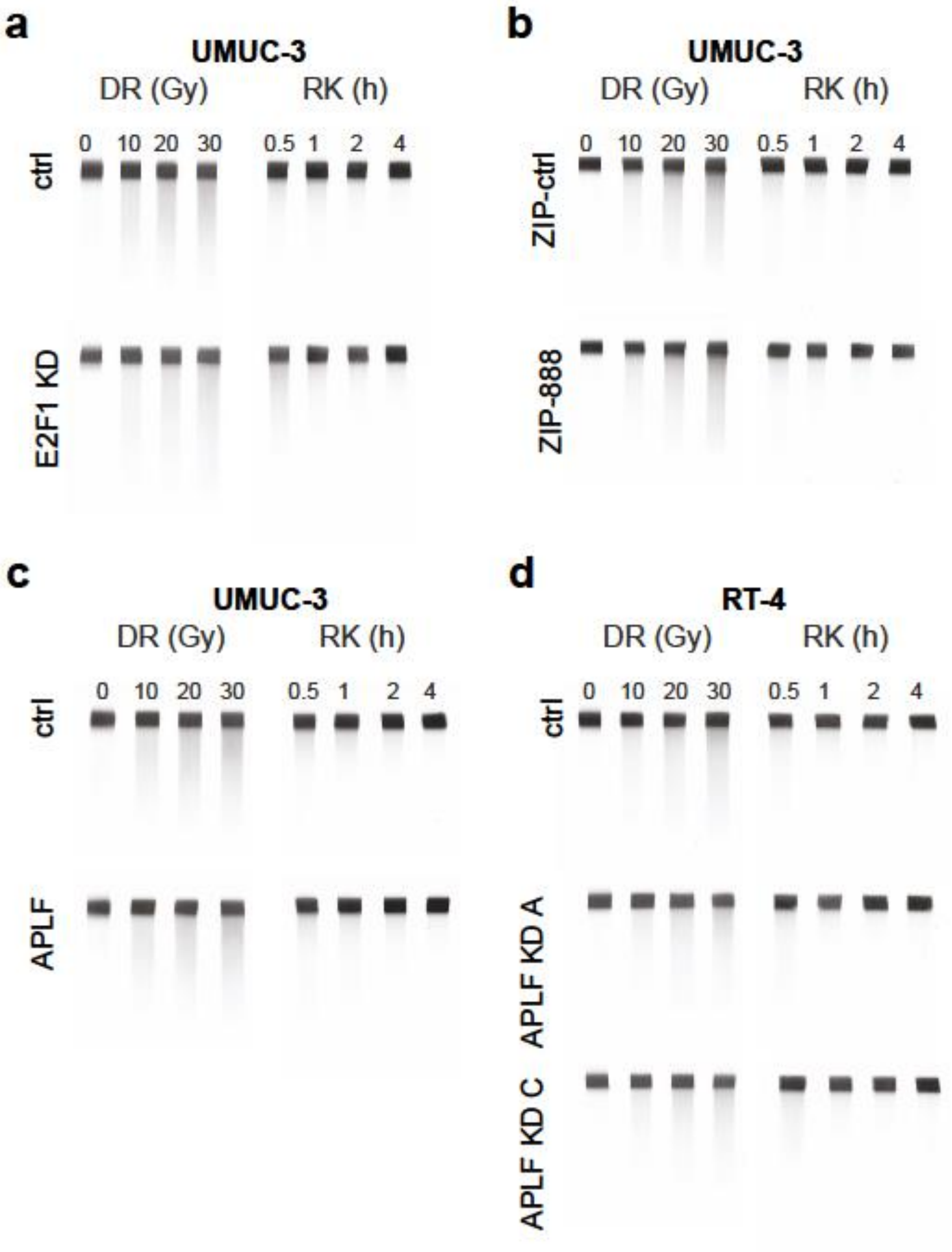

Fig. S4 Representative agarose gels depicting the fraction of DNA released (FDR) from the well into the gel for (a) UMUC-3 with E2F1 knockdown (KD), (b) miR-888-5p knockdown (ZIP-888), (c) APLF overexpression, and in (d) RT-4 with APLF knockdown (KD, clones A and C) versus their controls. Cells were irradiated with 0, 10, 20 or 30 Gy to construct the dose response (DR) curves. Repair kinetics (RK) was monitored at 0,5, 1, 2 or 4 hrs. The fraction from each sample was converted to DEQ and plotted as a function of time. DEQ values are extrapolated from the DR curves.
