# Supplementary material for: Rewiring E2F1 with classical NHEJ via APLF suppression promotes bladder cancer invasiveness
Source: J Exp Clin Cancer Res. 2019 Jul 8;38:292. doi: 10.1186/s13046-019-1286-9 (PMC6615232; doi:10.1186/s13046-019-1286-9)
Supplement: Supplementary file 6 — Figure S5. OS of BC patients with a high E2F1/high APLF tumor content upon elevated mRNA expression of the c-NHEJ complex factors. (PDF 2143 kb) [file 13046_2019_1286_MOESM6_ESM.pdf]

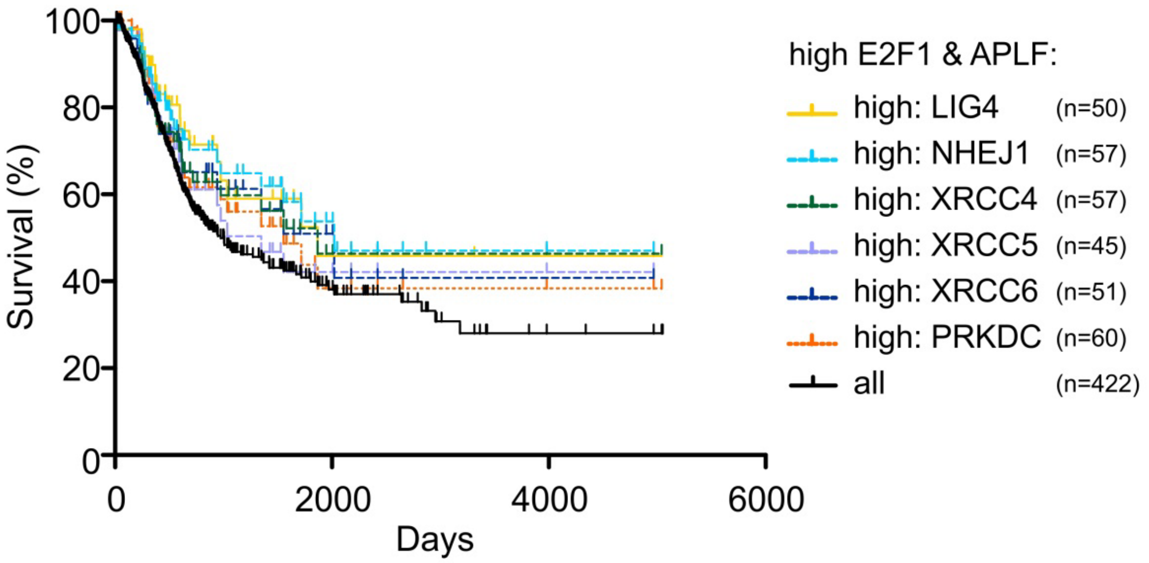

Fig. S5 OS of BC patients with a high E2F1/high APLF tumor content upon elevated mRNA expression of the c-NHEJ complex factors. Data were obtained and analyzed with TCGA genome browser.
