# Supplementary material for: Rewiring E2F1 with classical NHEJ via APLF suppression promotes bladder cancer invasiveness
Source: J Exp Clin Cancer Res. 2019 Jul 8;38:292. doi: 10.1186/s13046-019-1286-9 (PMC6615232; doi:10.1186/s13046-019-1286-9)
Supplement: Supplementary file 7 — Figure S6. OS of PAN Cancer cohort patients with a ‘high E2F1/high APLF/high DCLRE1C’ tumor signature. (PDF 117 kb) [file 13046_2019_1286_MOESM7_ESM.pdf]

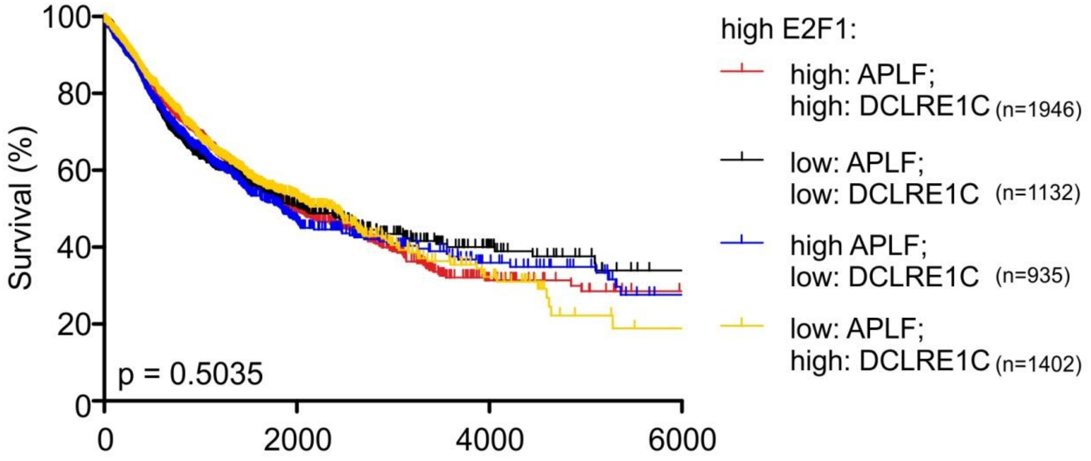

Fig. S6 OS of PAN Cancer cohort patients with a ‘high E2F1/high APLF/high DCLRE1C’ tumor signature (red) versus patients without this signature. There is no significant change in survival probability relative to ‘high E2F1/low APLF/low DCLRE1C’ (black), ‘high E2F1/high APLF/low DCLRE1C’ (blue), or ‘high E2F1/low APLF/high DCLRE1C’ (yellow) tumor signatures.
